# Prevalence of Antibiotic Resistance Among Common Clinical Bacterial Isolates From Kalar City, Iraq

**DOI:** 10.1155/ijm/2662976

**Published:** 2026-10-31

**Authors:** Rahman Karim Faraj

**Affiliations:** ^1^ Department of Chemistry College of Science University of Garmian Kalar Kurdistan Region Iraq garmian.edu.krd

**Keywords:** antibiotic resistance, clinical samples, gram negative bacteria, gram positive, VITEK 2

## Abstract

The rapid and precise detection of pathogens and their antimicrobial susceptibility profile is needed for infection control. This study is aimed at assessing the prevalence of multidrug‐resistant bacteria from clinical specimens at Zanko Central Laboratory, Kalar City, from October 7, 2024 to January 15, 2026, using the VITEK 2 compact system. A total of 1370 specimens were collected, of which 598 were positive for growth of clinically significant bacteria. The most frequently isolated gram‐negative bacteria were *Escherichia coli*, *Klebsiella pneumoniae*, and *Pseudomonas aeruginosa*, and the common gram‐positive isolates were *Staphylococcus haemolyticus*, *Enterococcus faecalis*, *Streptococcus agalactiae*, *Staphylococcus aureus*, and *Staphylococcus saprophyticus*. VITEK 2 system antimicrobial susceptibility testing already identified high resistance rates against cephalosporins, aminoglycosides, and fluoroquinolones, as well as some gram‐negative bacteria possessing extended‐spectrum beta‐lactamases (ESBL) and carbapenemase‐producing isolates. *P. aeruginosa* exhibited multidrug resistance to the majority of antibiotics. Among gram‐positive isolates, *E. faecalis* and *S. haemolyticus* were particularly resistant to cefoxitin, vancomycin, and erythromycin. These results highlight the rapid increase in the number of multidrug‐resistant bacteria and the immediate requirement for antimicrobial management programs, strict infection control practices, and routine monitoring. The VITEK 2 system was helpful in rapid and precise bacterial identification and quick susceptibility testing, which contributed to appropriate clinical action at the right time. ESBL production in *E. coli* and *K. pneumoniae* is not a reliable indicator for determining which isolates are most resistant. However, colistin resistance in *P. aeruginosa* indicates resistance to a broad range of antibiotics.

## 1. Introduction

The increasing rates of antimicrobial resistance pose an additional problem in healthcare systems since it might result in the ineffective management of infectious diseases along with increased morbidity and mortality [1]. One of the most concerning groups includes gram‐negative bacteria such as *Escherichia coli (E. coli*), *Klebsiella pneumoniae* (*K. pneumoniae*), *A. baumannii*, and *Pseudomonas aeruginosa* (*P. aeruginosa*) because of their multidrug‐resistant (MDR) nature [2]. Some gram‐positive organisms like *Staphylococcus saprophyticus* (*S. saprophyticus*), *Enterococcus faecalis* (*E. faecalis*), and Group B Streptococci also play a part in nosocomial infections for some sensitive populations like the elderly or the immunocompromised [3]. Effective management of infections and their control in patients requires timely diagnosis of these pathogens and their antibiotic susceptibility testing [2, 4].

The VITEK 2 Compact system is a pioneering device in the field of diagnostics that automates the processes of identifying microbes and testing for their susceptibility to antibiotics. Determination of antimicrobial susceptibility using minimum inhibitory concentrations with the VITEK 2 is well known and regarded as effective in guiding antibiotic therapy and monitoring resistance, with many studies noting its precision in tracking resistance trends and identifying both rare and common pathogens [5–7].

MDR bacteria have posed a significant public health challenge in the last few decades. The three top gram‐negative species that the World Health Organization poses a lot of concern about with respect to antibiotic resistance are *Acinetobacter baumannii* (carbapenems‐resistant), enterobacterales (third generation cephalosporins‐resistant), and enterobacterales (carbapenems‐resistant) [6, 8]. Along with complicating urinary tract infections, bloodstream infections, and pneumonia, these bacteria have critically reduced the effectiveness of antimicrobials as therapeutic options, greatly hindering the work of clinicians [9].

The existence of antibiotic‐resistant strains in various clinical settings has been emphasized in a number of prior research articles. The research performed in Iraq noted an increased occurrence of *E. coli* and *Staphylococcus aureus* in the clinical specimens. Moreover, *E. coli* showed a high level of resistance to cephalosporins and carbapenems [9]. In another study, also conducted in Tanzania, it was noted that the *S. aureus* isolates demonstrated nearly half carrying the *mec*A gene that resistance to commonly prescribed antibiotics; also it has various plasmids that indicate a complex resistance profile because of the dissemination of antibiotic‐resistance factors via horizontal gene transfer and chromosomal mutations caused by antibiotics [10].

As stated in Haji, Aka, and Ali (2021), the detection of those pathogens along with their antibiotic susceptibility profiles has to be performed accurately and promptly, as it helps in administering appropriate treatment and efficient infection control practices to reduce the spread of antibiotic resistance within the healthcare system. The wide clinical application of the VITEK 2 compact system stems from its proper and rapid identification of numerous bacteria and determination of their antimicrobial susceptibility [4].

As with other regions, the goal here is to evaluate an area′s multidrug resistance using an automated system for accurate diagnosis and infection management. It also assessed the VITEK 2 compact as a system diagnostic tool to determine if it has the potential to enhance the skills of personnel in diagnosing and managing infections caused by MDR organisms.

## 2. Materials and Methods

The data collection was to collect samples from patients who could frequently visit Zanko Medical Laboratory in the city of Kalar in the period between October 7, 2024 and January 15, 2026. Conducted along with the laboratory work, there was a survey among the patients of the lab. Patients of all ages and genders were included; a convenience sampling approach was used, whereby eligible specimens available during the study period were included according to the predefined study criteria. The samples were cultured using a manual routine protocol. A total of 1370 samples were collected, out of which 598 have been reported to be significant colonies. Specimens were excluded if they were insufficient in quantity, improperly collected or transported, contaminated before laboratory processing. Duplicate isolates from the same clinical episode were excluded according to the study protocol.

Our strategy was based on systematic approaches to employing the laboratory equipment using the routine technique of manual pipetting, working with media plates. For the identification, VITEK 2 compact (Biomerux/French) was applied after DensiCheck (Biomerux/French) was used to adjust and then transfer the microbe suspension into a dilution tube with saline. The crucial steps of the tests were performed using the following kits: AST‐N224 for gram‐negative lactose‐fermenting bacteria, AST‐N222 for nonlactose‐fermenting bacteria, AST‐592 for staphylococci, enterococci, and *Streptococcus agalactiae*, and AST‐ST03 for *S. agalactiae* and other streptococci.

Isolates were classified as infection strain bacteria that are MDR, ESBL‐producing for Enterobacteriaceae, and colistin‐nonfermenting *P. aeruginosa*, cefoxitin‐resistant staphylococci, and vancomycin‐resistant streptococci. Each kit contains antibiotics for microbial resistance. Additionally, each one consists of a set of antibiotics specific to an isolated group of a particular type. The system also provided the corresponding susceptibility ESBL‐related interpretation according to its validated software algorithms and interpretive criteria, and then the system classified as ESBL positive or negative.

Each specimen type was collected using an appropriate standardized procedure, approximately 20 mL of clean‐catch midstream urine, which was captured in sterile universal containers with lids. Vaginal specimens were collected by a healthcare professional using a sterile swab, and sputum specimens were collected in sterile, wide‐containers after appropriate patient instruction to obtain a deep respiratory specimen rather than saliva. All samples were correctly labeled and handled appropriately according to standard microbiological practices.

Antimicrobial susceptibility testing (minimum inhibitory concentration) was determined using the VITEK 2 automated system. The isolates were tested against a panel of antibiotics as outlined in each kit. The susceptibility results generated by the VITEK 2 compact system were used to determine the resistance profile of each isolate. MDR was assessed based on antimicrobial categories, and it was defined as acquired resistance to at least one agent in three or more antimicrobial categories [11].

## 3. Results and Discussion

The most commonly isolated samples were urine (522), followed by vaginal secretions (37), sputum (13), and other samples (26). This is because the urinary tract is the closest portal from the intestinal tract for infections to enter, and it is also sensitive to infection. Additionally, females represent a more frequent population (66.8%) among the samples, as they are more susceptible to infection because of their anatomy and physiology, as shown in Table 1. Our study findings revealed that the predominant bacterial isolates were *E. coli*, *K. pneumoniae*, and *P. aeruginosa* among gram‐negative bacteria, while *E. faecalis*, *S. haemolyticus*, *S. agalactiae*, *S. aureus*, and *S. saprophyticus* were frequently isolated. Because of their prevalence, we conducted susceptibility testing for common isolates in limited numbers, as shown in Tables 2 and 3.

**Table 1:** Appearance and isolation of all isolates.

Microbial name	Frequency	Patient sex		Samples			
		Female	Male	Urine	HVS	Sputum	Others
Gram‐negative bacteria	383	245	138	344	15	11	13
Gram‐positive bacteria	215	155	60	178	22	2	13
Total	598	400	198	522	37	13	26

**Table 2:** Resistance of common gram‐negative isolates to certain antibiotics presented in the kits.

Common bacterial species		Frequency of isolate	Cephalosporins						Penems			Aminoglycosides			Quinolones		Other antibiotics		
			Ampicillin	Amoxiclav	Piperacillin/tazobactam	Cefotaxime	Ceftazidime	Cefepime	Ertapenem	Meropenem	Imipenem	Amikacin	Gentamicin	Tobramycin	Ciprofloxacin	Fosfomycin	Nitrofurantoin	Trimethoprim/sulfamethoxazole	Colistin
*E. coli*	ESBL+	55	55	32	15	46	35	20	0	1	1	16	20		32	1	12	32	
	ESBL‐	35	24	15	5	5	4	4	0	2	2	4	21		15	3	6	21	
*K. pneumoniae*	ESBL+	13	13	6	0	11	9	4	0	0	0	1	5		3	3	11	9	
	ESBL‐	17	17	8	9	8	8	8	4	7	7	4	6		7	5	16	8	
			Ticarcillin	Ticarcillin/tazobactam	Piperacillin														
*P. aeruginosa*		21	21	16	19		14	14		16	16	15	17	17	20				3

**Table 3:** Resistance of common gram‐positive isolates to certain antibiotics presented in the kits.

Common bacterial species	Frequency of isolate	Antibiotics																
		Benzylpenicillin	Oxacillin	Ampicillin	Tigecycline	Linezolid	Rifampicin	Clindamycin	Teicoplanin	Erythromycin	Vancomycin	Gentamicin	Ciprofloxacin	Moxifloxacin	Tetracycline	Trimethoprim/sulfamethoxazole	Fusidic acid	Cefoxitin
*E. faecalis*	42			15	0	0			13	41	21	14	21		37			
*S. haemolyticus*	38	37	36		0	0	18	32	26	36	26	20	26	22	30	10	31	37
*S. agalactiae*	20				0	0		13			0			7	17			
*S. aureus*	18	18	11		0	0	5	13	8	13	10	0	8	10	13	3	2	10
*S. saprophyticus*	9	9	8		0	0	4	8	4	6	7	0	0	0	0	0	9	

About 268 (44.8%) common isolates among gram‐negative and gram‐positive were determined to be susceptible to antibiotics within the same minimum inhibitory concentration technique, 141 isolates of gram‐negative and 127 of gram‐positive, as shown in Tables 2 and 3.

The antimicrobial susceptibility testing showed that these pathogens exhibited varying degrees of resistance to commonly used antibiotics, with some strains demonstrating multidrug resistance. For instance, *E. coli* and *K. pneumoniae* exhibited high resistance to third‐generation cephalosporins and carbapenems, which are considered last‐line treatments for gram‐negative infections. These findings are consistent with the data reported in previous studies conducted in Iraq and Tanzania [9, 10].

ESBL‐producing *E. coli* were more resistant to cephalosporins, amikacin, ciprofloxacin, and nitrofurantoin than those without ESBL. Those without ESBL are more resistant to meropenem, imipenem, trimethoprim/sulfamethoxazole, gentamicin, and fosfomycin. But both of them were sensitive to ertapenem. Resistance to both cephalosporins and penicillins is also conferred by ESBLs [12] among other (nonbeta‐lactams) that are associated with the carriers of the ESBL genes [13]. Such mobile genetic elements frequently contain numerous resistance factors, resulting in coresistance to various antibiotic classes. Resistance to nitrofurantoin is less common and may be due to mutations in such genes as *nfsA* and *nfsB*, which code for the nitroreductases that activate the medication [14]. It is surprising that non‐ESBL *E. coli* is more resistant than ESBL *E. coli* to carbapenems (meropenem and imipenem) because these latter are usually expected to develop resistance to carbapenems, particularly through porin loss combined with either AmpC or ESBL production [15]. Nonetheless, some non‐ESBL *E. coli* resists the action of carbapenem antibiotics with the production of carbapenemases (e.g. bla_KPC, bla_NDM, bla_OXA‐48) or by overproduction of efflux pumps [16]. In non‐ESBL *E. coli*, gentamicin resistance is likely due to the action of aminoglycoside‐modifying enzymes (AMEs) such as aac(3)‐II and aac(6 ^′^)‐Ib common in non‐ESBL MDR strains [17].

Fosfomycin resistance is usually caused by mutations in the *murA* gene, overexpression of efflux pumps (*fosA*, *fosB*), or decreased drug uptake due to transporter mutations [18]. The presence of certain mechanisms of resistance to *E. coli* carbapenemase also enables some *E. coli* strains to be more resistant to fosfomycin. However, the sensitivity to Ertapenem within both populations points to the lack of substantial carbapenem resistance mechanisms. All of these resistances to the antibiotics are shown in Table 2 and observed in Figure 1.

**Figure 1:**
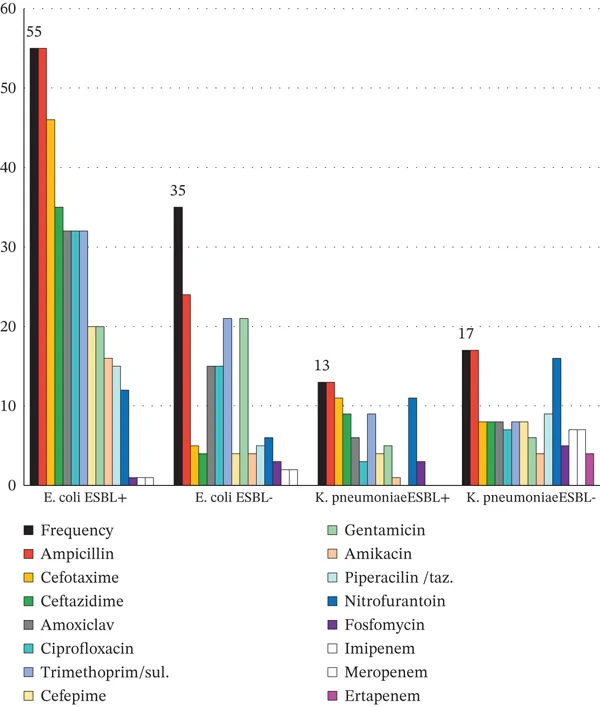
The resistance of *E. coli* and *K. pneumoniae* to antibiotics was presented.

Although *K. pneumoniae* without ESBL are more resistant than those with ESBL in a way isolates with ESBL are most resistant to ampicillin, cefotaxime, and ceftazidime among cephalosporins, and trimethoprim/sulfamethoxazole, whereas without ESBL, it is most resistant to ampicillin, amoxicillin/clavulanic acid, piperacillin/tazobactam, cefepime, and carbapenems among betalactams in addition to aminoglycosides, fluoroquinolones, and nitrofurantoin as shown in Table 2 and Figure 1. As in *E. coli*, ESBL enzymes are known to hydrolyze penicillins and cephalosporins, which explains why *K. pneumoniae* is resistant to ampicillin, cefotaxime, and ceftazidime [19]. Third‐generation cephalosporins became ineffective because of the presence of these enzymes. ESBL resistance is often attributed to it, followed by the development of coresistance on plasmids containing ESBL genes [20]. Some ESBL isolates respond to certain *β*‐lactam inhibitors like piperacillin/tazobactam, or sometimes carbapenems, with no other resistance mechanisms, which explains why it is sometimes seen in these isolates. Unlike non‐ESBL producing *E. coli*, the higher resistance in non‐ESBL *K. pneumoniae* over carbapenems, aminoglycosides, fluoroquinolones, and nitrofurantoin suggests that other resistance mechanisms apart from ESBL could stem from harboring carbapenemase genes (KPC, NDM, OXA‐48), or may possess a combination of porin loss and AmpC *β*‐lactamase [21], and may contribute to resistance to these antimicrobial agents. Gentamicin and amikacin are common aminoglycosides associated with resistance, whereas ciprofloxacin is a common fluoroquinolone. ESBL‐*K. pneumoniae* is strongly suspected to be a rapidly evolving hyper‐virulent strain in addition to resistance to nitrofurantoin. These two events are likely due to mutations in *nfsA* and *nfsB*, which stop the activation of the drug inside the bacterial cell [14].

The noted resistance of non‐ESBL *K. pneumoniae* to both amoxicillin/clavulanic acid and piperacillin/tazobactam may indicate the presence of either uncharacterized AmpC *β*‐lactamases or other *β*‐lactamase types that are resistant to inhibition by clavulanic acid or tazobactam [22]. Additionally, the greater resistance to cefepime in non‐ESBL isolates suggests the presence of either an AmpC‐type *β*‐lactamase or porin mutations, which reduce outer membrane permeability [23].

*P. aeruginosa* was the most resistant isolate to all other antibiotics, as shown in Table 2 and Figure 2. It is the most commonly isolated pathogen that is extensively resistant to all other antibiotics tested. One can expect this high level of resistance because of the intrinsic and acquired resistance mechanisms of *P. aeruginosa*, which is one of the most troublesome nosocomial pathogens. It differs from many other gram‐negative bacteria and possesses several mechanisms in its genome that enable it to exhibit natural broad‐spectrum resistance to various antibiotics.

**Figure 2:**
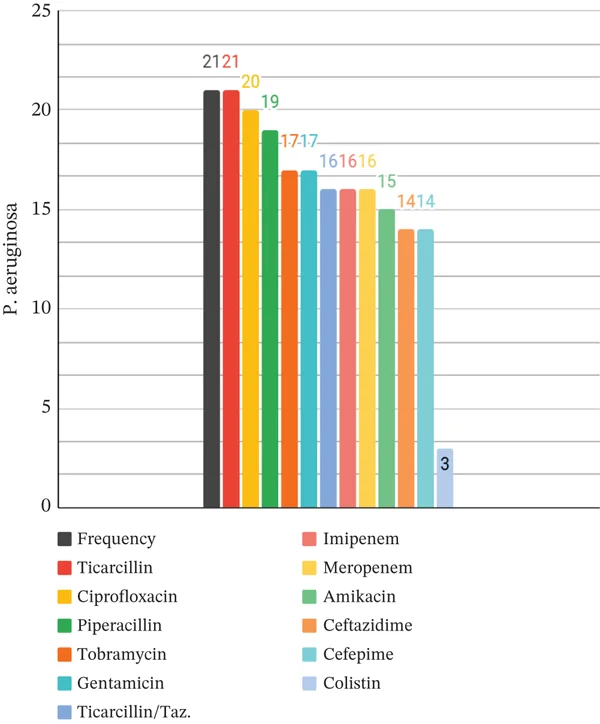
The resistance of 21 strains of *P. aeruginosa* to antibiotics was presented.

Moreover, *P. aeruginosa* acquires resistance because of mutations in the genes for DNA gyrase (*gyr*A, *gyr*B) and topoisomerase IV (*par*C, *par*E) that confer fluoroquinolone resistance [24]. There is also loss or modification of the *oprD* porin, which renders pseudomonas less sensitive to carbapenems such as imipenem and meropenem [25]. Together with the blaVIM and blaIMP genes that carry known carbapenemase resistance, AMEs, and extended‐broad spectrum beta‐lactamases that are known to be introduced by horizontal gene transfer [26].

*S. haemolyticus*, *S. aureus*, and *S. saprophyticus* demonstrated high resistance rates to benzylpenicillin, oxacillin, and cefoxitin; however, the latter does not indicate sensitivity to *S. saprophyticus*, as illustrated in Table 3 and clarified in Figure 3. This resistance is largely attributed to the acquisition of the *mecA* gene, which encodes an altered penicillin‐binding protein (PBP2a) with a reduced affinity for *β*‐lactams, resulting in methicillin‐resistant *Staphylococcus aureus* (MRSA) and methicillin‐resistant coagulase‐negative staphylococci (MR‐CoNS) [27].

**Figure 3:**
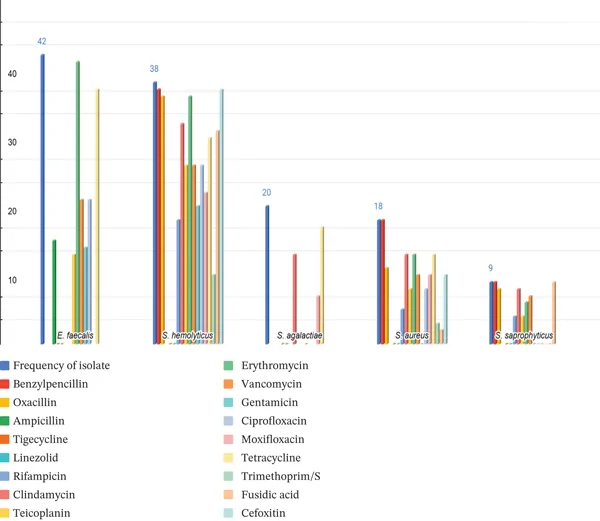
The resistance of common gram‐positive isolates to certain antibiotics.

The three staphylococci and *E. faecalis* showed high levels of resistance against vancomycin and teicoplanin, which are known to be mediated by acquisition of van genes, such as *vanA*, *vanB*, and *vanC*, which change the terminal amino acids of peptidoglycan from D‐alanyl‐D‐alanine to D‐alanyl‐D‐lactate or D‐alanyl‐D‐serine, reducing vancomycin binding affinity [28]. The emergence of vancomycin‐intermediate‐resistant *Staphylococcus aureus* (VISA) is a worrying development because these strains have thicker cell walls that trap vancomycin, leading to ineffective treatment [29].

Multi‐drug resistance due to a combination of different resistance factors is common among *S. haemolyticus*, which demonstrated this with macrolides (erythromycin), lincosamides (clindamycin), tetracycline, fluoroquinolones (ciprofloxacin), aminoglycosides (gentamicin), and rifampicin. This resistance is caused by the accumulation of many resistance genes, such as erm genes for macrolide‐lincosamide, tet genes for tetracycline, and *gyrA* mutations for fluoroquinolone [30].

The highest resistance to erythromycin and clindamycin was shown by *S. aureus*, *S. haemolyticus*, *S. agalactiae*, and *E. faecalis* as shown in Figure 3. This type of resistance is primarily attributed to erm genes that code for methylases, which modify the antibiotic binding site on the ribosome, resulting in cross‐resistance among macrolides, lincosamides, and streptogramins [31].

*E. faecalis* was resistant against gentamicin and known to be resistant against ciprofloxacin as well. Resistance to aminoglycosides is often linked with the production of enzymes that inactivate these antibiotics, known as AMEs. Mutations within the *gyrA* and *parC* genes, which code for subunits of DNA gyrase and topoisomerase IV, respectively, commonly result in the resistance of fluoroquinolones [32].

All isolates remained susceptible to linezolid and tigecycline. Linezolid inhibits bacterial protein synthesis by binding to the 50S ribosomal subunit, and resistance is rare but can occur through mutations in the 23S rRNA or ribosomal proteins. Tigecycline also prevents protein synthesis and continues to work with a number of MDR organisms [33].

*S. agalactiae* showed the lowest resistance overall, which was absent against vancomycin, linezolid, and tigecycline. Also, *S. saprophyticus* was responsive to fluoroquinolones and aminoglycosides, indicating the possibility of these antibiotic classes as an effective therapy for this organism, but it was highly resistant to most penicillins and vancomycin.

## 4. Conclusion

The multidrug resistance varies across different regions, particularly in Iraq. *P. aeruginosa* is the first bacterial species resistant among gram‐negative bacteria isolated from urine, followed by *E. coli* and *K. pneumonia*, which were also the two most prevalent in urine, in addition to vaginal secretions and sputum. Additionally, among gram‐positive *S. haemolyticus* is frequently observed and resistant to most groups of antibiotics, although it is typically regarded as a commensal organism residing on the skin and mucous membranes, followed by *S. aureus*, *S. saprophyticus*, and *E. faecalis*.

## Funding

No funding was received for this manuscript.

## Disclosure

I hereby confirm that all the figures and tables in the manuscript are mine. Any figures and images that are not mine have been included with the necessary permission for republication, which are attached to the manuscript.

## Ethics Statement

The project was approved by the local ethical committee at the University of Garmian. No animal studies are present in the manuscript. The author signed off on the ethical considerations′ approval.

## Conflicts of Interest

The author declares no conflicts of interest.

## Data Availability

The data that support the findings of this study are available on request from the corresponding author. The data are not publicly available due to privacy or ethical restrictions.
